# Synthesis, Spectral Characterization, and Biochemical Evaluation of Antidiabetic Properties of a New Zinc-Diosmin Complex Studied in High Fat Diet Fed-Low Dose Streptozotocin Induced Experimental Type 2 Diabetes in Rats

**DOI:** 10.1155/2015/350829

**Published:** 2015-12-09

**Authors:** Veerasamy Gopalakrishnan, Subramanian Iyyam Pillai, Sorimuthu Pillai Subramanian

**Affiliations:** ^1^Department of Biochemistry, University of Madras, Guindy Campus, Chennai 600 025, India; ^2^PG and Research Department of Chemistry, Pachaiyappa's College, Chennai 600 030, India

## Abstract

In view of the established antidiabetic properties of zinc, the present study was aimed at evaluating the hypoglycemic properties of a new zinc-diosmin complex in high fat diet fed-low dose streptozotocin induced experimental type 2 diabetes in rats. Zinc-diosmin complex was synthesized and characterized by various spectral studies. The complexation between zinc ions and diosmin was further evidenced by pH-potentiometric titrations and Job's plot. Diabetic rats were orally treated with zinc-diosmin complex at a concentration of 20 mg/kg b.w./rat/day for 30 days. At the end of the experimental period, the rats were subjected to oral glucose tolerance test. In addition, HOMA-IR and various biochemical parameters related to glucose homeostasis were analyzed. Treatment with zinc-diosmin complex significantly improved the glucose homeostasis in diabetic rats. Treatment with zinc-diosmin complex significantly improved insulin sensitivity, at least in part, through enhancing protein metabolism and alteration in the levels of muscle and liver glycogen. The assay of clinical marker enzymes revealed the nontoxic nature of the complex. Determination of renal tissue markers such as blood urea and serum creatinine indicates the renoprotective nature of the complex. These findings suggest that zinc-diosmin complex is nontoxic and has complimentary potential to develop as an antihyperglycemic agent for the treatment of diabetes mellitus.

## 1. Introduction

Type 2 diabetes mellitus (T2DM) is a heterogeneous disorder characterized by a progressive decline in insulin sensitivity followed by pancreatic *β*-cell dysfunction [1]. The interplay between insulin resistance and *β*-cell dysfunction remains elusive [2]. The prevalence of type 2 diabetes has been increasing alarmingly due to sedentary life style, obesity, and lack of exercise. Globally, around 382 million people had diabetes in 2013, with the prediction that this number could be doubled by 2035 [3]. This is a minimum number because, for each diagnosed case, there will be one undiagnosed case in first world and eight in the third world countries [4]. Several hypotheses have been proposed to describe the pathogenesis of type 2 DM; however the persistence of microvascular and premature macrovascular complications as the main cause of morbidity and mortality in people with diabetes is a constant reminder that our therapeutic and management strategies are inadequate for most patients [5].

Most of the currently used oral antihyperglycemic drugs such as sulphonylureas, *α*-glycosidase inhibitors, biguanides, and thiazolidinediones for the treatment of type 2 diabetes are often associated with adverse side effects or diminution in response after prolonged use [6]. Hence, the search for novel therapeutic agents without having long-term side effects and eliciting better antidiabetic activity at low concentration is necessitated. Many metallic elements play a crucial role in living systems. Some metal ions mimic or sensitize the action of insulin in rodent models as well as* in vitro* [7–9]. The intentional introduction of a metal ion into the biological system will be for either therapeutic or diagnostic purpose. The use of metal complexes as therapeutic drugs has become highly important over the last couple of decades resulting in a variety of exciting and valuable metallopharmaceutical drugs [10], having a wide range of structural-pharmacological activity relationships, and biochemical aspects of metals binding to cellular targets have lined a new possibility for scientific investigation in metal coordination. Before the discovery of insulin and its clinical use to treat diabetes mellitus, oral administration of sodium vanadate was reported for the treatment of DM in humans [11].

Besides vanadium, zinc also possesses significant insulin mimetic and insulin sensitizing effects [12, 13]. In 1980, Coulston and Dandona reported that zinc^2+^ ions stimulate* in vitro* rat adipocyte lipogenesis, which was similar to the action of insulin [14]. Zinc is an essential trace element which is distributed in the entire body (2 to 4 g), and a wide spectrum of proteins and transcription factors contain this metal ion. Zinc is necessary for the stability and function of more than 300 enzymes [15, 16]. It is well known that Zn plays an important role in the synthesis, storage, and secretion of insulin [17]. Chronic hyperglycemia causes increased urinary loss of zinc and decreased zinc levels in the body [18]. The decreased zinc level adversely affects the ability of the *β*-cells in the synthesis and storage of insulin [19, 20].

Zinc ions are expected to develop as a clinically useful metallopharmaceutical, like platinum-containing cisplatin and gold-containing auranofin as anticancer and antiarthritic drugs, respectively [21, 22]. However, designing of new zinc complexes requires special attention in terms of stability and structural properties under physiological conditions. Most of the zinc complexes so far investigated for their antidiabetic potential were inadequately absorbed in their inorganic form and required high doses that have been allied with undesirable side effects. In order to circumvent the toxicity and increase gastrointestinal tract absorption of zinc, several complexes have been formulated using ecologically derived nonnutrient plant secondary metabolites as organic ligands and studied for their antidiabetic properties [23–27].

Flavonoids are a diverse group of polyphenolic phytochemicals that are produced as secondary metabolites by various plants to protect them from environmental stress and injury [28]. A large number of flavonoids are known for their wide range of pharmacological effects on human health especially in quenching oxidative stress and preventing secondary complications [29]. Amongst flavonoids, flavones are known to chelate the metal ions with great affinity owing to the presence of *α*-hydroxycarbonyl group and their ability to quench the free radicals [30].

Diosmin (diosmetin-7-O-rutinoside) is one such naturally occurring bioflavone found profusely in the pericarp of citrus plants such as Meyer lemons and Buddha's finger fruits [31]. It was originally isolated from* Scrophularia nodosa* in 1925 [32] and later it was readily obtained by the dehydrogenation of hesperidin [33]. Diosmin is nontoxic [34] and reported to exhibit a wide range of pharmacological properties including antioxidant [35], antiproliferative [36], anti-inflammatory [37], and antidiabetic effects [38]. Having these beneficial as well as pharmacological aspects in view, in the present study, we have designed and synthesized a new zinc complex using diosmin as an organic ligand and evaluated its antidiabetic properties in high fat diet fed-low dose STZ induced experimental type 2 diabetes in rats.

## 2. Materials and Methods 

### 2.1. Chemicals

Zinc sulphate [ZnSO_4_·7H_2_O], diosmin (C_28_H_32_O_15_), and streptozotocin (STZ) were procured from the Sigma-Aldrich, St. Louis, USA. Ultra-sensitive ELISA kit for rat insulin assay was purchased from Crystal Chem Inc. Life Technologies, India. All the other chemicals and reagents used were of analytical grade.

### 2.2. Analytical Instruments

The IR spectral studies for both free diosmin and its metal complex were performed in solid state as pressed KBr pellets using a Perkin-Elmer FT-IR spectrophotometer (400–4000 cm^1^). Jeol Gc-mate mass spectrometer was used to obtain the mass spectrum of the complex. The ^1^H NMR and ^13^C NMR of diosmin as well as its complex were obtained at 300 MHz and 500 MHz, respectively, using a Bruker AM-500 instrument. The spectral analysis data were recorded without any modification for instrumental characteristics.

### 2.3. Zinc-Diosmin Complex Synthesis

Molar ratio method was followed in the synthesis of zinc-diosmin complex as previously reported for the synthesis of various zinc complexes with slight modifications [39–42]. Briefly, a DMSO solution (10 mL) containing zinc sulphate heptahydrate (0.287 g, 1 mM) was gradually added to a hot solution of DMSO (15 mL) containing diosmin (1.217 g, 2 mM). The resulting solution was dried in a pressurized rotary evaporator and the complex obtained was washed with diethyl ether and kept under vacuum over anhydrous calcium chloride.

### 2.4. pH-Potentiometric Titrations

The formation constant of the newly synthesized zinc-diosmin complex was determined by conducting potentiometric titrations using a ELICO Li 120 pH meter fitted with a glass and calomel electrode. The calomel electrode was initially calibrated as a hydrogen concentration probe by titrating known amounts of standardized HCl against standardized NaOH. A minimum of two independent titrations were carried out at 25°C ± 10°C for probe calibration. The protonation and stability constants were calculated from the pH data by using program Origin 8.5 lab [43]. For all titrations, the total sample volume was fixed as 20 mL and the ligand concentration was 1–10 mM. Six titrations were carried out for free diosmin alone and fifteen titrations defined the zinc complex equilibria at the pH range of 2–10 [44].

### 2.5. Experimental Animals

Male Albino rats of Wistar weighing around 160 to 180 g were procured from the Tamilnadu Veterinary and Animal Sciences University, Chennai, and were housed under standard husbandry conditions (12 ± 1 h light and dark cycle, relative humidity 55%  ±  10%). The animals were fed with balanced diet (Hindustan Lever Ltd., Bangalore, India) and water* ad libitum*. The rat pellet diet is composed of 55% nitrogen-free extract, 21% protein, 5% fat, and 4% fiber (w/w) with sufficient levels of vitamin and mineral. The experimental design was strictly conducted according to the ethical norms approved by the Ministry of Social Justices and Empowerment, Government of India and Institutional Animal Ethics Committee Guidelines (Approval number 02.01.2012), for the examination of experimental pain in conscious animals.

### 2.6. Acute Toxicity and Dosage Fixation Studies

Acute toxicity studies were performed as per OECD guidelines for testing of chemicals in normal rats. Graded doses (10, 20, 25, and 30 mg/kg b.w./rat) of zinc-diosmin complex dissolved in 5% DMSO were orally administered to rats. The changes in food consumption, fluid intake, psychomotor activities, and body weight gain and changes in skin, fur, eyes, salivation, diarrhea, and lethargy in rats were continuously monitored for a period of 30 days. Macroscopic examinations were also performed on vital organs. Oral administration of graded doses of zinc-diosmin complex (10, 20, and 30 mg/kg b.w./rat/day) for 30 days to determine the dose-dependent hypoglycemic effect in HFD-low dose STZ induced diabetic rats by monitoring the fasting blood glucose levels periodically to fix the effective dose of the zinc-diosmin complex for treatment.

### 2.7. Experimental Design

The rats were allocated into two dietary regimens by feeding either normal pellet diet (NPD) or high fat diet (HFD) for 2 weeks of dietary manipulation. HFD contains powdered NPD, 365 g/kg, lard, 310 g/kg, casein, 250 g/kg, cholesterol, 10 g/kg, vitamin and mineral mix, 60 g/kg, DL-methionine, 3 g/kg, yeast powder, 1 g/kg, and NaCl, 1 g/kg. After 2 weeks of HFD supplementation, Group II, Group III, and Group IV rats were injected with a single dose of STZ (35 mg/kg b.w./rat); control rats (Group I) fed with NPD were injected intraperitoneally with the same volume freshly prepared cold citrate buffer (pH 4.5, 0.1 mol/L) only [45]. After one week of STZ injection, rats having fasting blood glucose levels ≥ 300 mg/dL were considered as diabetic rats and chosen for further studies. The animals were divided into four groups, each comprising six rats as follows:

 Group I: normal control rats.
 Group II: HFD-STZ (i.p. 35 mg/kg b.w.) induced diabetic rats.
 Group III: HFD-STZ induced diabetic rats treated with zinc-diosmin complex (20 mg/kg b.w./rat/day) for 30 days.
 Group IV: HFD-STZ induced diabetic rats treated with metformin (200 mg/kg b.w./rat/day) for 30 days.

### 2.8. Oral Glucose Tolerance Test (OGTT)

On the day prior to sacrifice, oral glucose tolerance test (OGTT) was performed in all the groups of rats. Blood samples were collected from the lateral tail vein of rats deprived of food overnight. Successive blood sample was taken at 0, 30, 60, 90, and 120 minutes following the oral administration of 2 mg/kg b.w. of glucose solution [46].

### 2.9. Homeostasis Model Assessment of Insulin Resistance

As the insulin abnormality cannot be accurately detected by a single determination of insulin or glucose levels, the insulin resistance was evaluated by homeostasis model assessment of insulin resistance (HOMA-IR) as follows [47]:

(1) HOMA-IR=Fasting insulin level×Fasting blood glucose405.

### 2.10. Biochemical Parameters

At the end of the experimental period, overnight fasted rats were anaesthetized, using ketamine (80 mg/kg b.w./rat, i.p.) and sacrificed by cervical decapitation. Blood samples were collected with and without anticoagulant for separation of plasma and serum, respectively. For the estimation of glycogen [48], liver and muscle tissues were excised out, washed with ice-cold saline, and stored at −70°C and used. The basic biochemical parameters such as fasting blood glucose [49], glycosylated hemoglobin [50], plasma protein [51], blood urea [52], and serum creatinine [53] levels were estimated. Urine strips were used to detect the presence of glucose in urine. The levels of plasma insulin and C-peptide were assayed by ELISA using a rat insulin assay kit (Linco Research, St. Charles, MO, USA). The activities of clinical marker enzymes such as aspartate transaminase (AST) [54], alanine transaminase (ALT) [54], and alkaline phosphatase (ALP) [55] in serum were assayed. The activities of glycogen synthase [56] and glycogen phosphorylase [57] in liver tissues were also assayed.

### 2.11. Statistical Analysis

The results were expressed as mean ± SD of six rats per group and the statistical significance was evaluated by “one-way analysis of variance” (ANOVA) using the SPSS (version 16) program followed by least significance test. A value of *P* < 0.001, *P* < 0.01, and *P* < 0.05 was considered to indicate statistical significance.

## 3. Results 

**Scheme 1:**
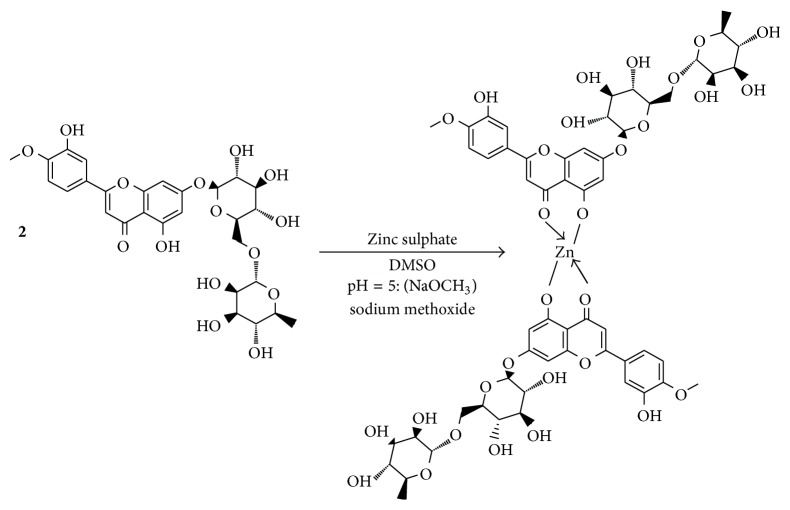
Structure of zinc-diosmin complex.

The zinc-diosmin complex (C_56_H_62_O_30_Zn) was synthesized by molar ratio method and obtained as a pale orange coloured powder after vacuum evaporation and the yield was about 76%. The schematic representation of complex synthesis is shown in Scheme 1.

**Figure 1:**
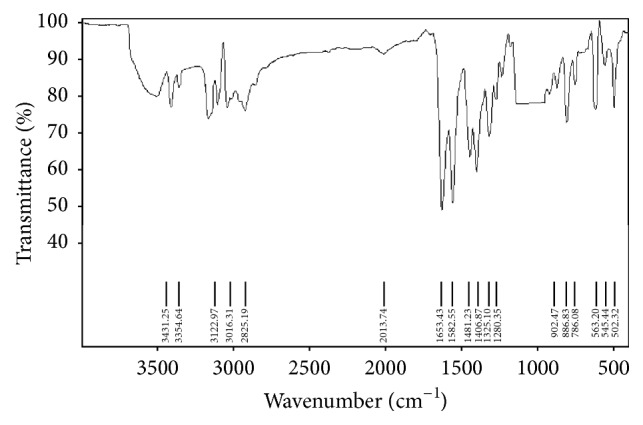
IR spectrum of zinc-diosmin complex.

**Figure 2:**
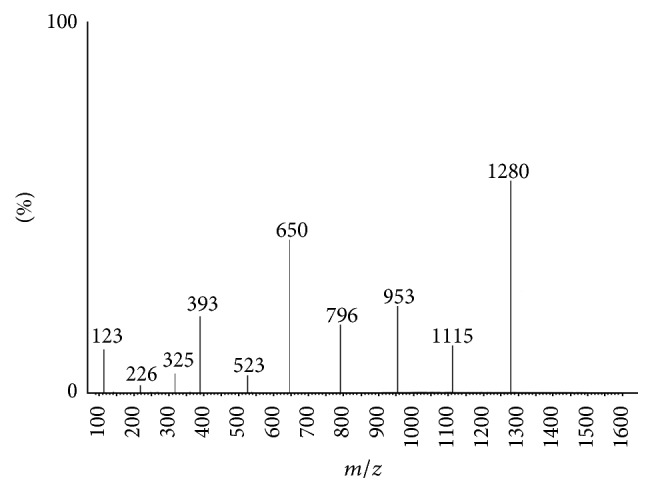
Mass spectrum of zinc-diosmin complex.

**Figure 3:**
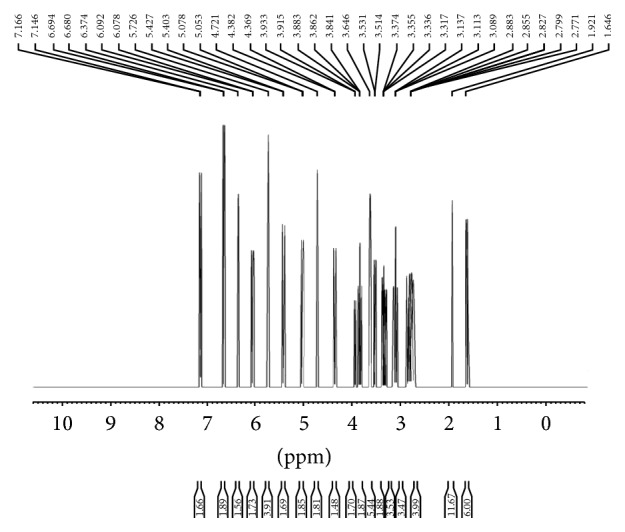
^1^H NMR of zinc-diosmin complex.

**Figure 4:**
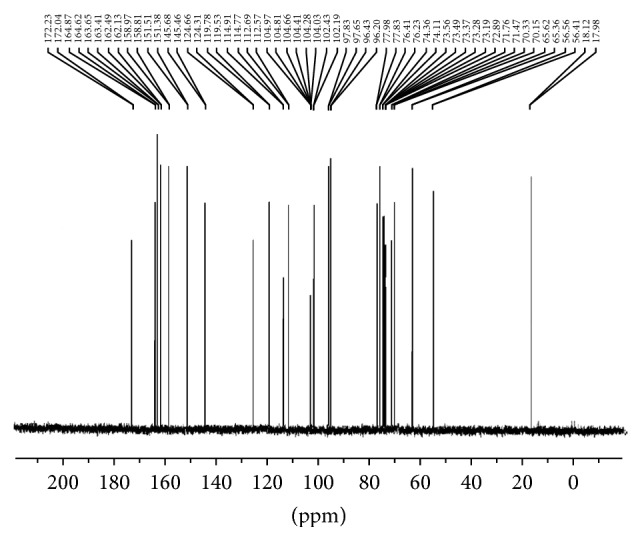
^13^C NMR of zinc-diosmin complex.

The IR spectral data of free diosmin is shown in Figure S1 [IR (KBr, *ν* cm^−1^) 3435 [–OH], 1676 (C=O)] (see Supplementary Material available online at http://dx.doi.org/10.1155/2015/ 350829). The IR spectrum of the zinc-diosmin complex is represented in Figure 1 [IR (KBr, *ν* cm^−1^) 1653 (C–O–M), 502 (M–O)].  Figure 2 represents the EI mass spectrum of complex (*m/z*): 1280. Elemental analysis calculated for C_56_H_62_O_30_Zn (1280) C, 52.53; H, 4.88. Found C, 52.47; H, 4.80. The ^1^H NMR and ^13^C NMR analysis of the zinc-diosmin complex are depicted in Figures 3 and 4, respectively. The ^1^H-NMR analysis of free diosmin (Figure S2) shows the resonances with coupling constants as follows (DMSO-d_6_, 300 Hz) *δ* H: 6.97 (d, 1H, *J* = 6.38 Hz), 6.68 (d, 1H, *J* = 6.82 Hz), 6.44 (s, 1H), 6.24 (d, 1H, *J* = 6.9 Hz), 5.84 (s, 2H), 5.68 (d, 1H, *J* = 6.74 Hz), 5.11 (d, 1H, *J* = 6.6 Hz), 4.83 (s, 2H), 4.22 (d, 1H, *J* = 6.9 Hz), 3.97 (d, 1H, *J* = 7.2 Hz), 3.84 (t, 1H, *J* = 8.8 Hz), 3.79 (s, 3H), 3.72 (d, 1H, *J* = 6.3 Hz), 3.54 (q, 2H), 3.31 (t, 2H, *J* = 10.2 Hz), 3.19 (m, 2H), 1.98 (s, 6H), 1.46 (s, 3H). The ^13^C-NMR analysis of free diosmin (Figure S3) shows the carbon resonances at (DMSO-d_6_, 500 Hz): 183.2, 164.8, 163.5, 162.6, 158.7, 151.1, 145.7, 124.5, 119.7, 114.1, 112.8, 104.7, 104.4, 102.0, 97.4, 96.3, 77.0, 76.2, 74.2, 73.3, 73.1, 73.0, 71.4, 70.3, 65.4, 56.9,18.

**Figure 5:**
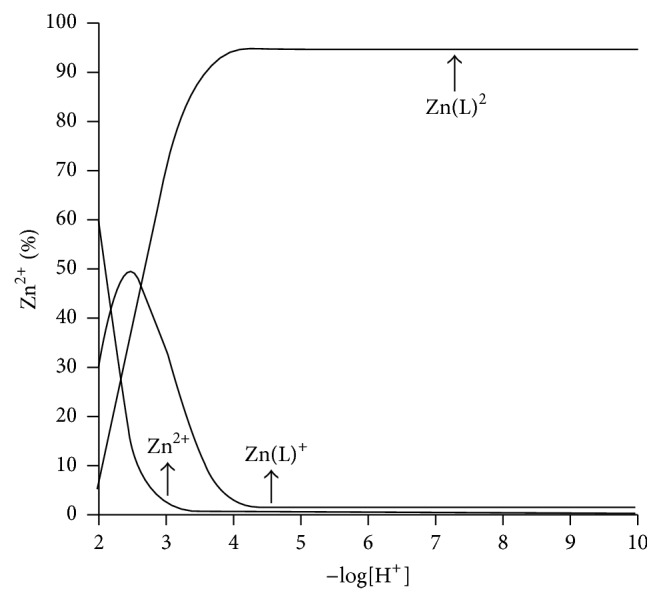
Potentiometric titration studies of zinc-diosmin complex.

Further, pH-potentiometric titrations were carried out (Figure 5) to understand the complex formation between zinc ion and diosmin molecules, by reported methods for other zinc complexes [58–60]. The stability constants of zinc-diosmin complex were listed below as (2), (3), (4), and (5). Potentiometric titrations of synthesized complex were reliable with the pattern of equilibria described earlier for synthesized metal complexes [61]. The analysis of the results was consistent with the subsequent equilibria:

(2) H++L−⇌HLlog⁡Kα=9.25

(3) Zn2++L−⇌ZnL+log⁡K1=9.86

(4) ZnL++L−⇌ZnL2log⁡K2=8.42

(5) Zn2++2L−⇌ZnL2log⁡β=18.28

**Figure 6:**
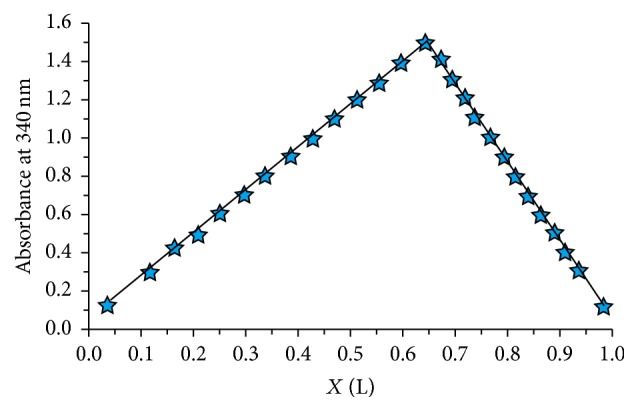
Job's plot measurements of zinc-diosmin complex.

Job's plot measurements were carried out to analyze the complexation ratio between zinc ion and ligand by altering the concentration of both the Zn^2+^ ions and ligand (Figure 6). The maximum point appears at the mole fraction of 0.65, close to the typical ligand mole fraction of 0.66 for a 2 : 1 ligand to metal complex, which is in accordance with our recent report [27].

**Figure 7:**
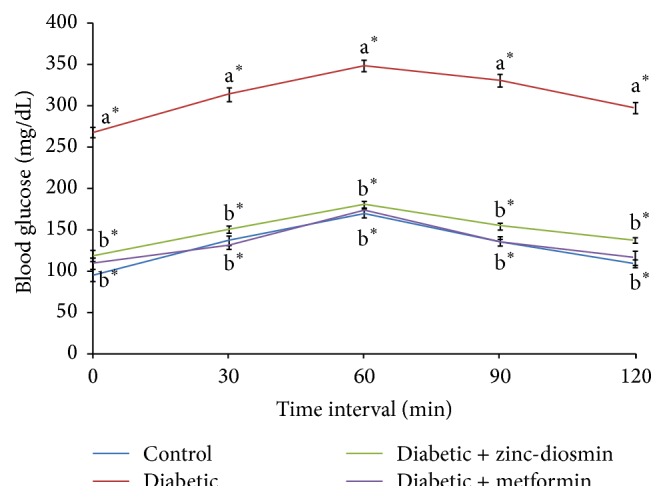
Effect of zinc-diosmin complex on oral glucose tolerance in the experimental groups of rats. Results are expressed as mean ± SEM [*n* = 6]. One-way ANOVA followed by post hoc test LSD was done. The results were compared with  ^a^Control rats and  ^b^Diabetic rats. Values are statistically significant at *P* < 0.05.

**Figure 8:**
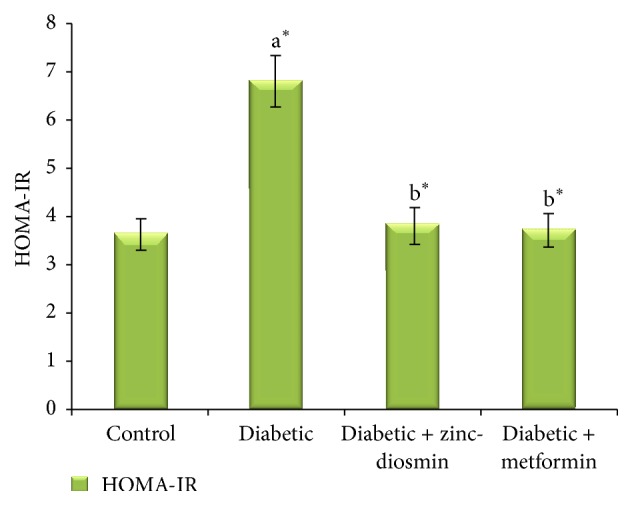
Effect of zinc-diosmin complex on insulin sensitivity in experimental groups of rats. Results are expressed as mean ± SEM [*n* = 6]. One-way ANOVA followed by post hoc test LSD was done. The results were compared with  ^a^Control rats and  ^b^Diabetic rats. Values are statistically significant at *P* < 0.05.

Figure 7 shows the effect of zinc-diosmin complex on the levels of blood glucose in certain durations after the oral administration of glucose (2 g/kg b.w.) in control and experimental groups of rats. The fasting blood glucose level of diabetic rats was significantly higher when compared to control group of rats. Subsequent to oral glucose load, blood glucose levels in HFD-STZ-diabetic rats reached a peak at 60 min and did not come back to the normal basal level over the next 60 min. In control rats, the blood glucose level reached the maximum peak at 60 min after an oral glucose load and then it was gradually reverted back to near normal after 120 min. Diabetic rats treated with zinc-diosmin complex as well as metformin resulted in a significant reduction in concentration of blood glucose at 0 (fasting), 30, and 60 min compared with untreated HFD-STZ-diabetic rats. In addition, zinc-diosmin complex as well as metformin treated diabetic rats showed blood glucose levels returned to basal value at 120 min after the oral glucose load. HOMA-IR values of control, diabetic, and diabetic rats treated with zinc-diosmin complex are depicted in Figure 8. Diabetic rats showed a significant elevation of HOMA-IR that was decreased significantly upon treatment with oral administration of complex as well as metformin.

Table 1 depicts the levels of fasting blood glucose, glycosylated hemoglobin, and plasma insulin and the inference for the presence of urine sugar in all groups of experimental rats. Diabetic rats showed significantly elevated levels of fasting glucose and glycosylated hemoglobin and the levels were significantly reduced in zinc-diosmin complex treated diabetic rats. Plasma insulin and C-peptide levels in the diabetic rats are markedly reduced when compared with control rats whereas these altered levels were significantly improved in diabetic rats treated with zinc-diosmin complex as well as metformin. Urine sugar was present in diabetic rats. However, after 30 days of treatment with zinc-diosmin complex as well as metformin, urine sugar was no longer detected.

The liver and muscle glycogen content in control and experimental groups of rats are represented in Table 2. The glycogen contents in liver and muscle tissues were significantly decreased in diabetic rats when compared with control rats and the levels were brought back near normal after treatment with zinc-diosmin complex. The diabetic rats treated with metformin also showed significantly improved glycogen content in liver and muscle tissues.  Table 3 shows the levels of plasma protein, blood urea, and serum creatinine in control and experimental groups of rats. The increased levels of renal tissue markers such as urea and creatinine and reduced levels of plasma protein in diabetic rats were significantly altered in zinc-diosmin treated diabetic rats. Metformin treated diabetic rats also showed similar improvement in the levels of blood urea, serum creatinine, and plasma protein. The activities of pathophysiological enzymes such as AST, ALT, and ALP in the serum of control and experimental groups of rats are shown in Table 4. Diabetic control rats showed elevated activities of these enzymes when compared with control rats and the elevated activities were reduced significantly in diabetic rats treated with zinc-diosmin complex. Similar observations were recorded in the diabetic rats treated with metformin.

The activities of glycogen synthase and glycogen phosphorylase in the liver tissue of control and experimental groups of rats are represented in Table 5. Diabetic rats showed a significant decline in the glycogen synthase activity and a concomitant increase in the activity of glycogen phosphorylase. Oral administration of zinc-diosmin complex as well as metformin to HFD-STZ diabetic rats restored the activities of glycogen synthase and glycogen phosphorylase.

## 4. Discussion 

The binding mode of diosmin to zinc ions was studied by comparing the IR spectrum of free diosmin with the spectrum of zinc-diosmin complex. The *α*-hydroxycarbonyl group of flavonoids was reported as the preferential site for the binding of the metal ions. The peaks observed at 1676 and 3435 (*ν* cm^−1^) correspond to the presence of carbonyl and hydroxyl groups in the free ligand, respectively. These bands have undergone a shift to a lower frequency [1653 and 3354 (*ν* cm^−1^)] after complexation, indicating the coordination of the hydroxyl and carbonyl groups with the zinc ion. The nature of bonding was further confirmed by a newly formed band at 502 cm^−1^ in the spectrum of the complex, which is tentatively assigned to M–O vibration. In both free diosmin and zinc-diosmin complex, the peaks around 1500 cm^−1^ are due to the presence of C=C stretching. This observation is in accordance with an earlier report of Ai et al. [62].

Molecular weight of the synthesized zinc-diosmin complex was confirmed by the molecular ion peak [M^+^] at* m/z* = 1280. Various fragments C_50_H_52_O_25_Zn, C_44_H_42_O_20_Zn, C_39_H_40_O_14_Zn, C_33_H_29_O_10_Zn, C_26_H_17_O_8_Zn, and C_9_H_5_O_3_Zn show the peaks at* m/z* = 1115, 953, 796, 650, 523, and 226, respectively, in mass spectral analysis. Thus, the mass spectra data suggest that the molar fraction of zinc and diosmin in the complex is in the ratio of 1 : 2, which concurs with an earlier report [63].

The ^1^H NMR studies were carried out on a Bruker AM 500 instrument using d_6_-DMSO as the solvent. The ^1^H NMR spectrum of the free diosmin showed 32 proton peaks. Diosmin exhibits a singlet signal at 4.82 ppm corresponding to two aromatic free hydroxyl groups, whereas, in the case of zinc-diosmin complex, instead of four-hydroxyl proton signals only two signals were observed. This clearly evidenced that the deprotonated hydroxyl group is involved in the chelation. Both the free diosmin and zinc-diosmin complex show a peak around 1.4 ppm and 3.7 ppm due to the presence of –CH_3_ and –OCH_3_ groups. The aromatic proton peaks were also observed around 5.6–6.9 ppm (6 H). Similarly, the proton signals for saccharide protons were observed in the range of 3.1–5.1 ppm. There are no appreciable changes in the rest of the signals in the synthesized complex [64].

The zinc-diosmin complex as well as the free diosmin was also characterized by means of ^13^C NMR spectroscopy. The ^13^C NMR spectra showed similar characteristic features of both free diosmin and zinc-diosmin complex. The peaks assigned to the aromatic carbons were found in the range of 119–164 ppm. The peaks around 65–77 ppm and peak at 18 ppm indicate the presence of glucose moiety carbons and the CH_3_ carbons, respectively. Likewise, the –OCH_3_ peak was observed around 56 ppm. The peaks around 96, 104, and 114 ppm denote the presence of –CH group present in the benzene ring. Similarly, the presence of the C=O signal at about 183 ppm in the spectrum of free diosmin and the absence of this signal in the spectrum of the zinc-diosmin complex along with the appearance of a signal at a lower value (172 ppm) supported the evidence that the zinc-diosmin complex was formed through carbonyl carbon. Thus, both the ^1^H NMR and ^13^C NMR spectroscopic data support the proposed structure of the synthesized complex. The NMR analyses obtained were in accordance with an earlier report [65].

Increasing prevalence of type 2 diabetes worldwide has underscored the necessity of developing an efficient therapeutic agent without any adverse side effect. Recently, increasing attention has been given to the role of certain trace elements in the pathogenesis of diabetes mellitus and its secondary complications. Total zinc content in the pancreatic *β*-cells is among the highest in the body and alterations in the Zn^2+^ level have been found to be associated with diabetes [66]. In order to improve the bioavailability and reduce the toxicity of zinc, several zinc complexes have been synthesized and studied for their insulinomimetic and insulin sensitizing effects. In the present study, diosmin a naturally occurring flavonoid glycoside was used as a ligand to synthesize a new zinc complex. Diosmin is known to regulate glucose metabolism by enhancing the activities of glycolytic enzymes by stimulating the insulin production from the remnant *β*-cells of pancreas [67].

Based on the dose-dependent effect of zinc-diosmin complex on the levels of fasting blood glucose concentration, 20 mg/kg b.w./rat/day for 30 days was fixed as the optimum dosage for evaluating the antidiabetic properties of the complex. High fat diet fed-low dose streptozotocin induced experimental type 2 diabetes in rats is an ideal animal model as it closely resembles the clinical and metabolic characteristics of human type 2 diabetes and widely used for pharmacological screening [67, 68]. Therefore, it is used in the present study to evaluate the antidiabetic properties of zinc-diosmin complex.

Blood glucose control is an important component in delaying or preventing acute or long-term diabetic complications. Though insulin resistance is the initiating pathogenic factor in type 2 diabetes, *β*-cell failure is accountable for insulin deficiency and impaired glucose tolerance to explicit type 2 diabetes [69]. In the present study, an observed low level of plasma insulin in HFD-STZ induced rats indicates perturbations in *β*-cell function. Furthermore, the degree of hyperinsulinemia and insulin resistance was substantiated from HOMA-IR values. HFD-STZ rats showed significantly higher HOMA-IR values. Hemoglobin in the circulation binds to glucose under physiological conditions by nonenzymatic, irreversible covalent bonding to form glycosylated hemoglobin (HbA1c). In diabetes, the level of HbA1c is significantly elevated because of increased glycation of hemoglobin due to persistent hyperglycemia. The level of HbA1c reflects the average blood glucose level over the past 3 months. Thus, HbA1c levels serve as a golden marker for the diagnosis as well as prognosis of diabetes mellitus. The significant reduction in the level of HbA1c in zinc-diosmin complex treated diabetic rats may be due to the maintenance of normal glucose homeostasis which is further evidenced from the results of OGTT and FBG. Significant reduction in the HOMA-IR indices of zinc-diosmin complex treated rats also indicates the efficacy of the complex in restoring insulin sensitivity.

Previous studies indicate that oral administration of diosmin at a concentration of 100 mg/kg b.w./rat/day for 30 days significantly reduced the blood glucose levels and controlled the secondary complications in STZ-nicotinamide induced experimental diabetes in rats. Though the dosage fixed in the present study for the treatment of diabetes is 20 mg/kg b.w. of zinc-diosmin complex, the results are comparable with the efficacy of the diosmin used in the earlier studies [38, 70].

Under normal physiological conditions, the excess glucose is stored in the form of glycogen mainly in liver and muscle tissues. However, the levels of intracellular glycogen deposition were reduced in diabetes because of diminished insulin activity, as insulin facilitates intracellular glycogen storage by inhibiting the activity of glycogen phosphorylase and stimulating the activity of glycogen synthase [71]. The glycogen contents in liver and muscle tissues were reduced in HFD-STZ induced type 2 diabetic rats. However, upon treatment with zinc-diosmin complex, the glycogen content was increased indicating the improved insulin sensitivity in the liver and muscle tissues.

The presence of elevated levels of intracellular enzymes such as transaminases and serum alkaline phosphatase in the circulation is the most sensitive and dramatic indicators of hepatic cell injury. During tissue damage, these soluble enzymes are leached out from the cells and their activities were found to be increased in the serum [72]. Elevated levels of these enzymes were also an indication of cellular damage and functional integrity loss of the cell membranes. A strong association exists among serum ALT level and HOMA-IR scores but not for the AST level and it has been indicated as an interpreter of type 2 diabetes in humans [73]. The increased activities of hepatocellular marker enzymes such as AST, ALT, and ALP in diabetic rats were reduced significantly upon treatment with zinc-diosmin complex indicating the nontoxic and tissue protective nature of the complex.

The metabolism of proteins is abnormal in HFD-STZ induced type 2 diabetes due to enhanced insulin resistance and muscle wasting. This ultimately results in profound increase in protein catabolism and significant reduction in protein synthesis [74]. The imbalance between anabolism and catabolism of proteins causes remarkable effects in the metabolic functions of renal tissues. Disturbances in renal function cause elevated levels of blood urea and serum creatinine, because urea is the main end product of protein catabolism and creatinine is a byproduct formed by the breakdown of creatine and phosphocreatine, which are considered as energy storage compounds in muscle tissues. The elevated levels of blood urea and serum creatinine suggest abnormality in the renal tissue to excrete urea and creatinine. The levels of these markers were significantly decreased in complex treated diabetic rats indicating the beneficial effect of the complex in ameliorating diabetes associated renal complications.

## 5. Conclusion 

In conclusion, the present study reveals the formation of complex between zinc ions and diosmin and its characterization by various spectral studies, pH-potentiometric titrations, and Job's plot. Oral administration of zinc-diosmin complex to HFD-STZ induced type 2 diabetic rats restored the activities of hepatocellular marker enzymes exemplifying the nontoxic nature of the complex. In addition, zinc-diosmin complex treatment leads to improved insulin sensitivity, at least in part, through improving protein metabolism and alterations in the levels of muscle and liver glycogen. These findings suggest that zinc-diosmin complex has complimentary potency to develop as an antihyperglycemic drug for the treatment of diabetes and its secondary complications. Further studies are in progress to elicit the molecular mechanisms involved in the antihyperglycemic action of zinc-diosmin complex in maintaining normoglycemia in type 2 diabetes mellitus.

## Supplementary Material

FT-IR of diosmin: In order to study the binding mode of diosmin with the zinc ion, the FT-IR spectrum of the zinc-diosmin complex was compared with the FT-IR spectrum of the free ligand. The FT-IR spectrum of the diosmin was presented as Fig S1. A sharp peak observed around 1675 cm^−1^ is owing to the presence of free carbonyl group present in the free ligand. Likewise, the broad band around 3435 cm^−1^ and a medium band around 1490 cm^−1^ corresponds to the presence of hydroxyl groups and C=C stretching in the free ligand respectively.
^1^H NMR and ^13^C NMR of the free diosmin: The ^1^H NMR and ^13^C NMR of the free diosmin as well as Zn-diosmin complex were recorded in DMSO-d_6_ and the data are reported along with their possible assignments in the discussion section. The protons and carbons were found in the expected regions. Fig S2 and Fig S3 shows the ^1^H NMR and ^13^C NMR spectra of the free diosmin.

## Figures and Tables

**Table 1:** The levels of blood glucose, glycosylated hemoglobin (HbA1c), plasma insulin, and urine sugar in control and experimental groups of rats.

Groups	Blood glucose	HbA1c	Insulin	C-peptide	Urine sugar
Control	95.75 ± 6.04	4.51 ± 0.29	15.41 ± 0.27	0.25 ± 0.023	Nil
Diabetic control	268.16 ± 9.97^a*∗*^	10.87 ± 0.43^a*∗*^	10.28 ± 0.43^a*∗*^	0.11 ± 0.007^a*∗*^	+++
Diabetic + Zn-diosmin	118.69 ± 4.57^b*∗*^	6.23 ± 0.32^b*∗*^	13.07 ± 0.55^b#^	0.17 ± 0.010^b*∗*^	Nil
Diabetic + metformin	109.47 ± 5.40^b*∗*^	5.79 ± 0.97^b*∗*^	13.82 ± 0.47^b#^	0.19 ± 0.012^b*∗*^	Nil

*Units are expressed as* mg/dL for blood glucose, % hemoglobin for HbA1c, *μ*U/mL for plasma insulin, and pmol/mL for plasma C-peptide; +++ indicates more than 2% sugar.

Results are expressed as mean ± SEM [*n* = 6]. One-way ANOVA followed by post hoc test LSD was done. Values are statistically significant at ^#^ *P* < 0.01; ^*∗*^ *P* < 0.001. The results were ^a^compared to control rats and ^b^compared to diabetic rats.

**Table 2:** Effect of Zn-diosmin complex on glycogen content in liver and muscle tissues of control and experimental groups of rats.

Groups	Liver glycogen	Muscle glycogen
Control	42.29 ± 1.83	9.48 ± 0.30
Diabetic control	21.33 ± 0.72^a*∗*^	3.99 ± 0.18^a*∗*^
Diabetic + Zn-diosmin	36.14 ± 1.43^b*∗*^	7.32 ± 0.28^b*∗*^
Diabetic + metformin	36.59 ± 1.25^b*∗*^	8.39 ± 0.31^b*∗*^

*Units are expressed as* mg of glucose/g wet tissue for glycogen.

Results are expressed as mean ± SEM [*n* = 6]. One-way ANOVA followed by post hoc test LSD was done. Values are statistically significant at ^*∗*^ *P* < 0.001. The results were  ^a^compared to control rats and ^b^compared to diabetic rats.

**Table 3:** Effect of Zn-diosmin complex on plasma protein and blood urea and serum creatinine levels in rats after 30-day treatment.

Groups	Plasma protein	Blood urea	Serum creatinine
Control	8.88 ± 0.19	25.28 ± 0.90	0.60 ± 0.02
Diabetic control	6.46 ± 0.1^a*∗*^	45.10 ± 1.56^a*∗*^	1.38 ± 0.02^a*∗*^
Diabetic + Zn-diosmin	7.42 ± 0.12^b*∗*^	29.33 ± 0.96^b*∗*^	0.75 ± 0.01^b*∗*^
Diabetic + metformin	8.21 ± 0.17^b*∗*^	27.33 ± 0.75^b*∗*^	0.68 ± 0.02^b*∗*^

*Units are expressed as* g/dL for plasma protein, mg/dL for blood urea, and serum creatinine.

Values are given as mean ± SEM for groups of six rats in each. One-way ANOVA followed by post hoc test LSD was done. Values are statistically significant at ^*∗*^ *P* < 0.001. The results were  ^a^compared to control rats and ^b^compared to diabetic rats.

**Table 4:** Effect of Zn-diosmin complex on levels of activities of AST, ALT, and ALP in the serum of control and experimental groups of rats.

Groups	AST	ALT	ALP
Control	71.06 ± 2.28	21.27 ± 0.63	72.45 ± 0.85
Diabetic control	131.72 ± 2.26^a*∗*^	45.24 ± 1.64^a*∗*^	149.09 ± 1.45^a*∗*^
Diabetic + Zn-diosmin	83.94 ± 2.28^b*∗*^	24.51 ± 1.01^b*∗*^	93.85 ± 2.05^b*∗*^
Diabetic + metformin	71.39 ± 1.90^b*∗*^	21.14 ± 0.94^b*∗*^	71.75 ± 1.74^b*∗*^

*Enzyme activities are expressed as* AST and ALT – l moles of pyruvate liberated/h/mg of protein, ALP – l moles of phenol liberated/min/mg of protein.

Results are expressed as mean ± SEM [*n* = 6]. One-way ANOVA followed by post hoc test LSD was done. Values are statistically significant at ^*∗*^ *P* < 0.001. The results were  ^a^compared to control rats and ^b^compared to diabetic rats.

**Table 5:** The activities of glycogen synthase and glycogen phosphorylase in liver tissues of control and experimental groups of rats.

Groups	Glycogen synthase	Glycogen phosphorylase
Control	829.61 ± 20.42	513.56 ± 25.67
Diabetic control	439.45 ± 15.64^a*∗*^	783.63 ± 22.63^a*∗*^
Diabetic + Zn-diosmin	667.13 ± 16.66^b*∗*^	659.16 ± 14.69^b*∗*^
Diabetic + metformin	690.96 ± 18.84^b*∗*^	633.48 ± 17.39^b*∗*^

*Units are expressed as* *μ*moles of UDP formed/h/mg protein for glycogen synthase and *μ*moles Pi liberated/h/mg protein for glycogen phosphorylase.

Results are expressed as mean ± SEM [*n* = 6]. One-way ANOVA followed by post hoc test LSD was done. Values are statistically significant at ^*∗*^ *P* < 0.001. The results were  ^a^compared to control rats and ^b^compared to diabetic rats.
