# Supplementary material for: Synthesis, Spectral Characterization, and Biochemical Evaluation of Antidiabetic Properties of a New Zinc-Diosmin Complex Studied in High Fat Diet Fed-Low Dose Streptozotocin Induced Experimental Type 2 Diabetes in Rats
Source: Biochem Res Int. 2015 Dec 9;2015:350829. doi: 10.1155/2015/350829 (PMC4689890; doi:10.1155/2015/350829)
Supplement: Supplementary file 1 — FT-IR of diosmin: In order to study the binding mode of diosmin with the zinc ion, the FT-IR spectrum of the zinc-diosmin complex was compared with the FT-IR spectrum of the free ligand. The FT-IR spectrum of the diosmin was presented as Fig S1. A sharp peak observed around 1675 cm−1 is owing to the presence of free carbonyl group present in the free ligand. Likewise, the broad band around 3435 cm−1 and a medium band around 1490 cm−1 corresponds to the presence of hydroxyl groups and C=C stretching in the free ligand respectively. 1H NMR and 13C NMR of the free diosmin: The 1H NMR and 13C NMR of the free diosmin as well as Zn-diosmin complex were recorded in DMSO-d6 and the data are reported along with their possible assignments in the discussion section. The protons and carbons were found in the expected regions. Fig S2 and Fig S3 shows the 1H NMR and 13C NMR spectra of the free diosmin. [file 350829.f1.pdf]

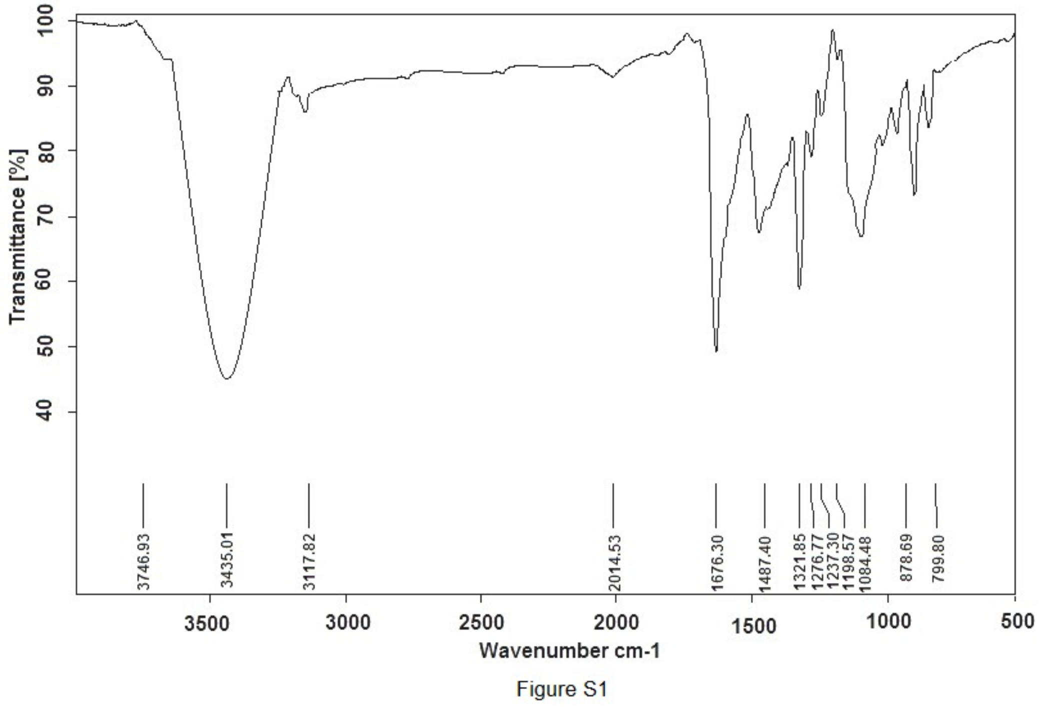

Figure S1: IR spectrum of Diosmin

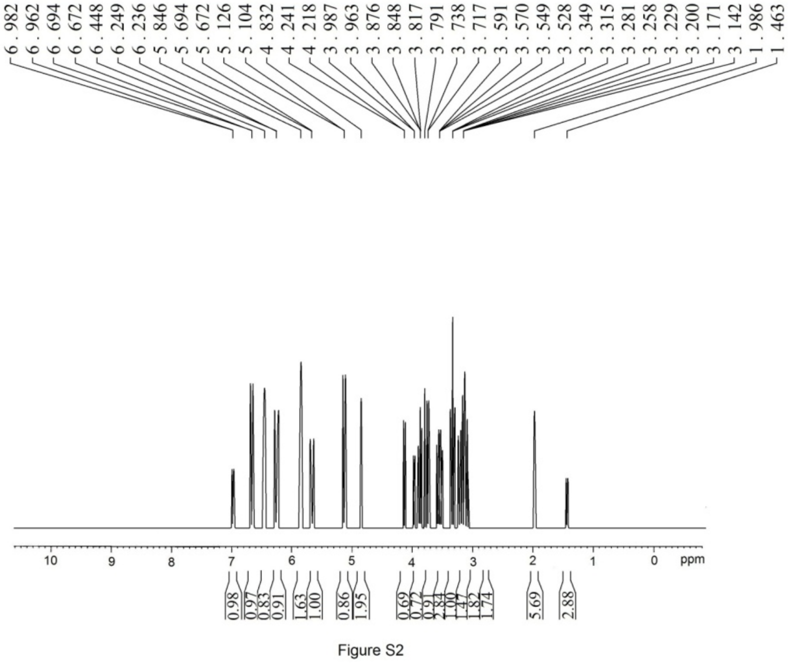

Figure S2: ^{1}H NMR of Diosmin

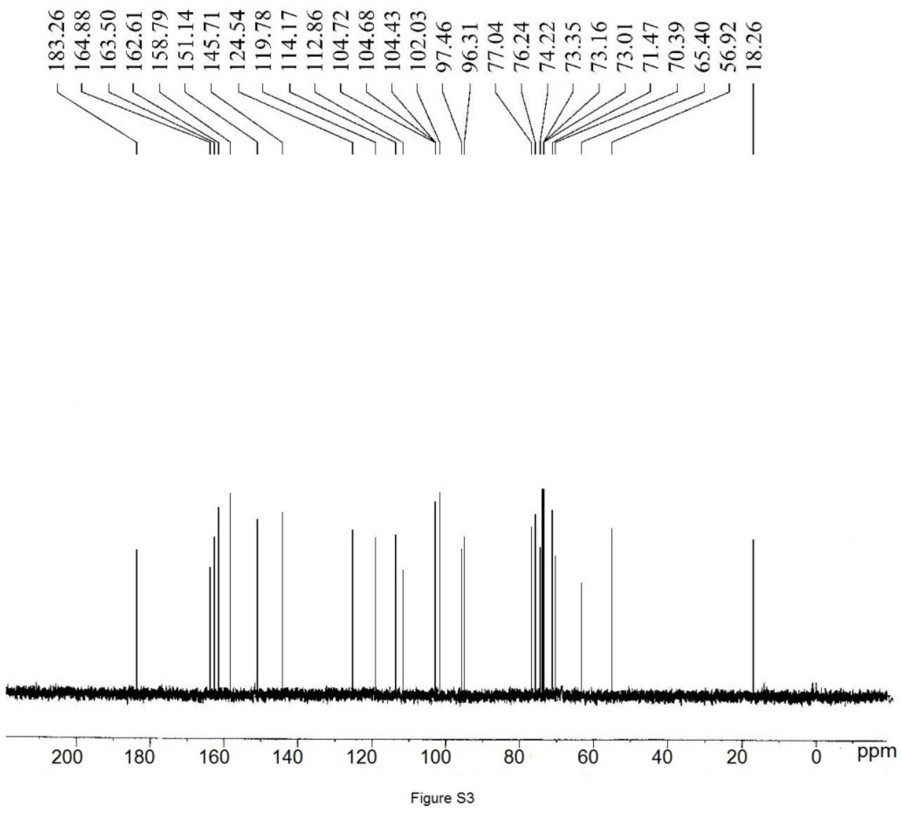

Figure S3: ^{13}C NMR of Diosmin
